# Performance of ultrasound in detecting fetal hypospadias during pregnancy: a pooled analysis

**DOI:** 10.1016/j.eclinm.2025.103091

**Published:** 2025-02-01

**Authors:** Qiang Zhang, Hongsong Chen, Chong Wang, Zhenmin Liu, Guanghui Wei, Zhicheng Zhang, Xing Liu

**Affiliations:** a Department of Urology Children's Hospital of Chongqing Medical University, National Clinical Research Centre for Child Health and Disorders, Ministry of Education Key Laboratory of Child Development and Disorders, Chongqing, 400014, China; b Chongqing Key Laboratory of Structural Birth Defect and Reconstruction, Chongqing, 400014, China; c Chongqing Key Laboratory of Children Urogenital Development and Tissue Engineering, Children's Hospital of Chongqing Medical University, Chongqing, 400014, China; d China International Science and Technology Cooperation Base of Child Development and Critical Disorders, Children's Hospital of Chongqing Medical University, Chongqing, 400014, China

**Keywords:** Hypospadias, External genitalia, Fetal, Ultrasound, Positive predictive value, Meta-analysis

## Abstract

**Background:**

Opinions regarding the role and value of ultrasound in the diagnosis of prenatal hypospadias are diverse. Although hypospadias is not a fatal malformation, a higher degree of severity exerts a significant impact on children's physiology and psychology. On the other hand, hypospadias may be accompanied by accompanying anomalies, syndromic conditions, and fetal growth restriction (FGR). This study aimed to systematically assess the value of ultrasound in the prenatal diagnosis of hypospadias.

**Methods:**

In the systematic review and meta-analysis, we searched PubMed, Web of Science, Scopus, Embase, and China National Knowledge Infrastructure (CNKI) databases until June 30, 2024. The key terms included: hypospadias, ultrasound, and prenatal diagnosis. Studies were assessed following the eligibility criteria, and the data from the included studies were extracted through a standardized protocol. The primary outcomes focused on the positive predictive value (PPV), accuracy, sensitivity, and specificity of ultrasound for detecting fetal hypospadias. The Quality Assessment of Diagnostic Accuracy Studies-2 (QUADAS-2) scale was used to assess the risk of bias. The PPV was calculated using the bootstrap method. When five or fewer studies were pooled, a random-effect model based on the Hartung-Knapp-Sidik-Jonkman (HKSJ) method was used as a meta-analysis strategy to evaluate the overall effect. Sensitivity and specificity were pooled using a Summary Receiver Operating Characteristic (SROC) curve. The protocol was registered with PROSPERO (CRD42024586840).

**Findings:**

A total of nine studies with 600 cases were included in the systematic review. The pooled median gestational age of the included studies was 27.64 ± 3.15 weeks. The overall PPVs of two-dimensional ultrasound (2DUS) in diagnosing fetal hypospadias and external genital malformations were 81% (95% confidence interval (CI): 77%–85%) and 88% (95% CI: 86%–91%), respectively. Upon in-depth analysis of the five studies, the accuracy of 2DUS combined with three-dimensional ultrasound (3DUS) was 84% (95% CI: 78%–89%), and that of 2DUS alone was 74% (95% CI: 69%–78%). The difference between the two methods was 10%. The pooled sensitivity of 2DUS combined with 3DUS in diagnosing fetal hypospadias was 86% (95% CI: 79%–93%), the specificity was 77% (95% CI: 69%–86%), and the area under the curve (AUC) was 0.86 (95% CI: 0.83–0.89).

**Interpretation:**

This is the first diagnostic meta-analysis to comprehensively evaluate the detection of fetal hypospadias using ultrasound during pregnancy, indicating that ultrasound is indeed of significant value in the prenatal diagnosis of hypospadias. More research is needed to validate and enhance current research findings and offer more comprehensive guidance for future clinical practice.

**Funding:**

This work was supported by the 10.13039/501100012669Natural Science Foundation Project of Chongqing, Chongqing Science and Technology Commission (CSTB2022NSCQ-MSX1001) and the Program for Youth Innovation in Future Medicine, 10.13039/501100004374Chongqing Medical University (W0109).

**Research in context**

**Evidence before this study**

While ultrasound serves as the main prenatal screening approach for hypospadias, it has certain false positive and false negative rates in the diagnostic performance and difficulty in determining the optimal time for diagnosis. To fully understand the existing research landscape, we searched the PubMed, Web of Science, Scopus, Embase, and China National Knowledge Infrastructure (CNKI) databases using key terms (“hypospadias”, “ultrasound”, “prenatal diagnosis”) to identify all relevant articles up to June 30, 2024, without any limitations on language, publication date, publication status, type of literature source, or research field, and found no relevant systematic reviews.

**Added value of this study**

To our knowledge, this is the first diagnostic meta-analysis to comprehensively evaluate ultrasound for fetal hypospadias detection during pregnancy. Our findings revealed a high diagnostic performance in detecting fetal hypospadias when combining two-dimensional and three-dimensional ultrasounds (2DUS + 3DUS). The optimal timing for diagnosis remains uncertain. The prenatal correlation between fetal hypospadias and fetal growth restriction (FGR) emphasizes the significance of perinatal factors, guiding prenatal screening for pregnant women at high risk and enhancing the overall understanding and management of fetal hypospadias.

**Implications of all the available evidence**

The findings highlight the crucial role of ultrasound in diagnosing fetal hypospadias. Future research under multidisciplinary collaboration, multi-imaging modalities combination, and cross-regional cooperation should be conducted to improve the current diagnostic landscape and yield more insights into the optimal timing for diagnosis.

## Introduction

Hypospadias, a common penile anomaly, exhibits regional differences in incidence. Nevertheless, the overall incidence is approximately within the range of 0.2–0.4%, and notably, it has shown an upward trend in recent years.^1^^,^^2^ Incomplete fusion of urethral folds results in an abnormal location of the ventral urethral opening of the penile.^3^ Urethral suture fusion gradually forms from the proximal to the distal of the genital tubercle (GT). Any hindrances may result in abnormal urethral openings during urethral suture fusion or remodeling of the GT. Simultaneously, hypospadias is frequently accompanied by ventral curvature and shortening of the penile.^4^, ^5^, ^6^ Ventral penile dysplasia restricts normal penile extension to some extent, shortening its length and adversely affecting the patient's sexual and reproductive functions in adulthood. Although hypospadias has a high incidence rate, prenatal diagnosis remains a significant challenge in clinical practice.

Visualizing genitals via ultrasound is crucial for fetuses suspected of having hypospadias. With its repeatability, low cost-effectiveness, and non-invasiveness, ultrasound has become the main approach for screening fetal malformations, allowing for monitoring fetal growth, development, and organ morphology.^7^ Ultrasound evaluation of fetal external genitalia can determine fetal sex and confirm the normal development of external genitalia.^8^ The main ultrasound features of suspected fetal hypospadias are as follows: (i) short/small penile; (ii) abnormal morphology of the distal penile (blunted penile tip), often accompanied by excessive dorsal prepuce; (iii) ventral penile curvature (chordee); (iv) a fan-shaped urinary stream emerging from the abnormal opening of the urethra instead of the tip of penile, which is difficult to observe. Vibration acoustic stimulation of the fetus can initiate urination to assist in diagnosis.^7^^,^^9^^,^^10^ The commonly used two-dimensional ultrasound (2DUS) and three-dimensional ultrasound (3DUS) techniques have advantages and disadvantages in diagnosing fetal hypospadias. Although 2DUS shows relatively poor clarity for some fine structures, 3DUS can serve as a complementary technique to provide a more intuitive three-dimensional image of the fetal external genitalia, facilitating more accurate observation of details such as the urethral opening location by allowing a multi-planar analysis and volumetric visualization, thereby possessing greater significance.^11^^,^^12^ While ultrasound serves as the main prenatal screening method for hypospadias, it exhibits certain false positive and false negative rates in the diagnostic performance and difficulty in determining the starting time of diagnosis.^13^ This may result from the small fetal GT and the intricate intrauterine environment (including amniotic fluid volume, fetus position, and status of the tissues surrounding the GT, etc.), which make subtle changes in the GTs easily overlooked. A meta-analysis is essential to assess and address these concerns and to provide a more comprehensive understanding of the overall diagnostic performance of ultrasound in diagnosing fetal hypospadias, including positive predictive value (PPV), accuracy, sensitivity, specificity, and area under the curve (AUC).

## Methods

### Study registration and search strategy

This study followed the Preferred Reporting Items for Systematic Reviews and Meta-Analyses (PRISMA) guidelines^14^ and its protocol was registered with PROSPERO (CRD42024586840) (www.crd.york.ac.uk/prospero). PubMed, Web of Science, Scopus, Embase, and CNKI databases were searched to identify all relevant articles up to June 30, 2024, without any limitations on language, publication date, publication status, type of literature source, or research field. The following search terms were used in the literature search: (fetal and (hypospadias OR genital diseases OR penile diseases)) AND (prenatal diagnosis OR intrauterine diagnosis OR antenatal diagnosis OR prenatal screening OR antenatal screening OR fetal diagnosis OR fetal screening) AND (ultrasonography OR medical sonography OR ((ultrasonographic OR ultrasonic OR ultrasound) AND imaging) OR diagnostic ultrasound OR ultrasonic diagnosis). The detailed search terms for each database and the corresponding study numbers are presented in Table S1.

### Eligibility criteria

For this meta-analysis, the following inclusion criteria were: (1) randomized controlled trials (RCTs), cohort studies, case-control studies, and cross-sectional studies that evaluated the value of ultrasound in diagnosing fetal hypospadias and external genital anomalies; (2) the outcome measures included the PPV, true positives (TP), false positives (FP), false negatives (FN), and true negatives (TN) of ultrasound in diagnosing fetal hypospadias and external genital anomalies; (3) the main diagnostic criteria included the ultrasound features of fetal hypospadias and external genital anomalies: (i) blunted penile tip; (ii) ventral penile curvature; (iii) a fan-shaped urinary stream; (iv) short/small penile^7^^,^^10^^,^^15^; (4) studies published with full texts accessible (can be comprehensively analyzed and evaluated). The exclusion criteria were as follows: (1) case reports (analyzing individual cases, lack representativeness); (2) magnetic resonance imaging (MRI) studies (this study focused on ultrasound to diagnose fetal hypospadias; therefore, MRI-related studies were excluded); (3) review articles (mainly comprehensive expositions of existing research, not original data).

### Data extraction and quality assessment

QZ and SHC screened and identified relevant studies based on the eligibility criteria. Subsequently, CW and MZL extracted the data and evaluated the quality of the included studies following the Quality Assessment of Diagnostic Accuracy Studies-2 (QUADAS-2) scale.^16^ In case any discrepancies remain, they are resolved by seeking a third opinion (HGW, CZZ, XL). Moreover, the following information from each included study: probe brand, study design, number of cases, languages, median maternal age, median gestational age at diagnosis, region of study, fetal karyotype, the ultrasound findings of hypospadias, other findings, PPV, accuracy, TP, FP, FN, and TN of fetal hypospadias and external genital malformation.

### Data analysis

The primary outcomes focused on the diagnostic performance of ultrasound for fetal hypospadias, covering PPV, accuracy, sensitivity, specificity, and AUC. Secondary outcomes were the optimal gestational age for diagnosing fetal hypospadias, the correlation between fetal hypospadias and fetal growth restriction (FGR), and the common ultrasound manifestations for diagnosing fetal hypospadias.

The TP, FP, FN, and TN values were collected to calculate accuracy, sensitivity, specificity, and PPV. The Hartung-Knapp-Sidik-Jonkman (HKSJ)^17^^,^^18^ method and DerSimonian-Laird (DL)^19^ method offer feasible analytical alternatives in statistical analysis. Given the small number of studies incorporated in the meta-analysis, we adopted the random-effect model based on the HKSJ method as a meta-analysis strategy to evaluate the overall effect more rationally. When handling between-study heterogeneity and unbalanced sample sizes, the HKSJ method exhibits greater robustness in confidence interval calculation and can better control the error rate.

Considering the expected negative correlation between sensitivity and specificity, we employed the bivariate random effects model. Subsequently, we utilized logit-transformed sensitivity and specificity to construct the Summary Receiver Operating Characteristic (SROC) curve.^20^ Pooled accuracy and 95% CI were calculated to assess the diagnostic performance of 2DUS combined with 3DUS and 2DUS alone. Sensitivity analyses were conducted by excluding individual studies to determine the robustness of the results. Additionally, heterogeneity was examined using the *I*^2^ statistic (≥50% or higher indicates moderate heterogeneity), and the sources of the heterogeneity were explored using the Knapp-Hartung method.^21^ The 95% confidence intervals (CIs) of PPVs were calculated using the bootstrap method (1000 resampling). Publication bias was evaluated through visual inspection of funnel plot symmetry and implementation of Egger's test. When evaluating AUC values, excellent corresponds to 0.9–1, good corresponds to 0.8–0.9, general corresponds to 0.7–0.8, and poor corresponds to AUC <0.7. The rating quality of evidence was evaluated using the GRADE approach.^22^ Statistical analysis was performed using R (version 4.1.2) and STATA/MP 17.0 software (StataCorp, College Station, TX, USA). *P* < 0.05 were considered significant.

### Ethics

Due to the nature of this study with publicly available and published aggregated data, no ethical approval was required.

### Role of funding source

This work was supported by the Natural Science Foundation Project of Chongqing, Chongqing Science and Technology Commission (CSTB2022NSCQ-MSX1001) and the Program for Youth Innovation in Future Medicine, Chongqing Medical University (W0109).

## Results

### Study characteristics

**Fig. 1:**
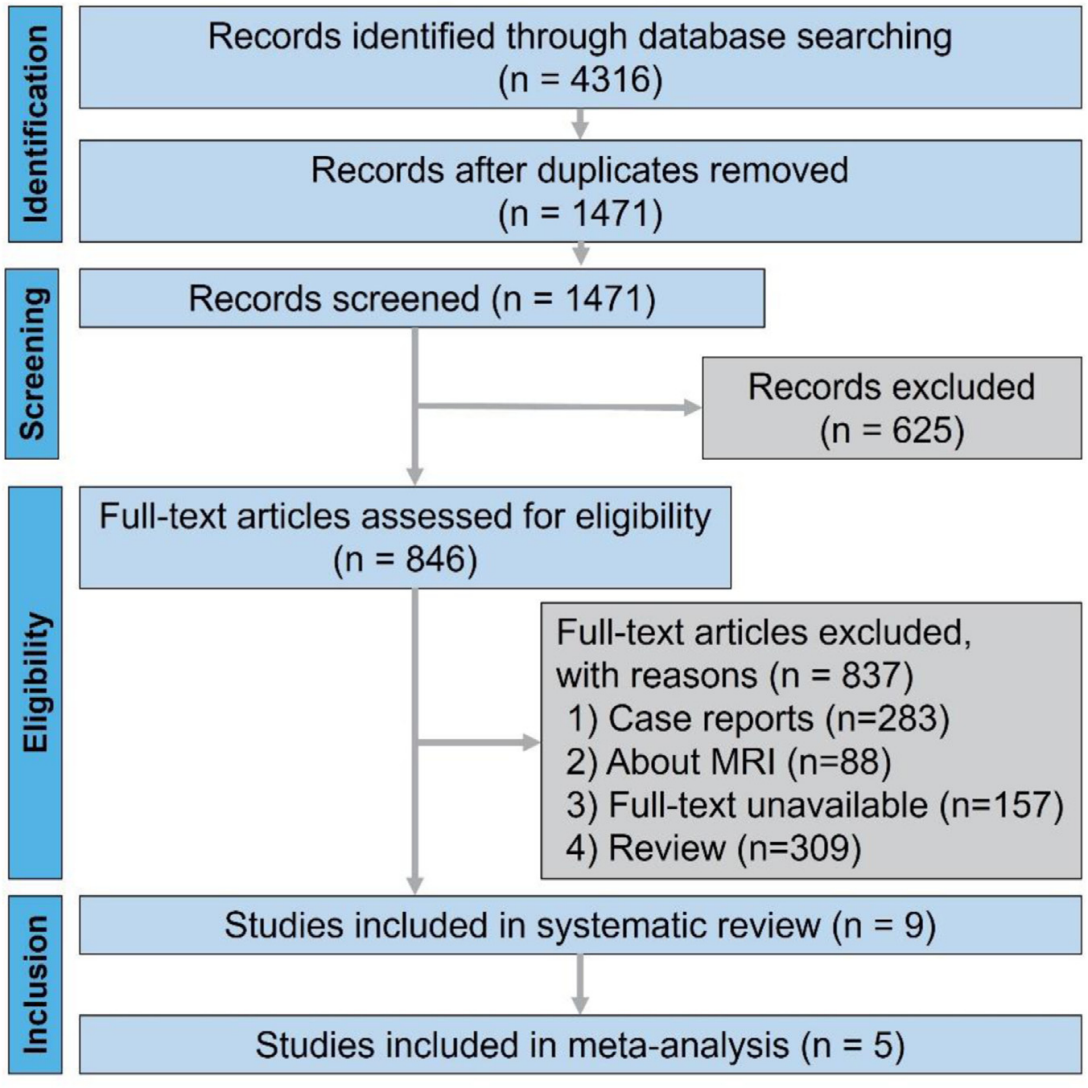
Flowchart of literature search. Abbreviation: MRI, magnetic resonance imaging.

Fig. 1 shows the process of literature search, screening, and inclusion. A total of 4316 articles were identified through a literature search, and nine studies with 600 cases were included in the systematic review. The characteristics of the nine studies in the systematic review are presented in Table 1.^11^^,^^13^^,^^23^, ^24^, ^25^, ^26^, ^27^, ^28^, ^29^ All nine studies included in the systematic review are cohort studies. Geographically, five were done in China, one in the United States, two in France, and one in Turkey. The probe brands used in the included studies were Philips, Samsung, and General Electric. Although karyotype analysis plays an important role in diagnosing chromosomal abnormalities related to fetal hypospadias, only a few studies performed fetal karyotype detection. The included studies indicated that the pooled median gestational age was 27.64 ± 3.15 weeks, within the middle and late stages of pregnancy. Given that the data were predominantly sourced from specific high- and middle-income regions, the results might not accurately reflect the performance of ultrasound in diagnosing fetal hypospadias in more routine or resource-limited settings.

**Table 1:** Characteristics of the nine studies included in the systematic review.

References	Probe brand	Study design	Languages	Number of cases	Median maternal age (y)	Median gestational age at diagnosis (w)	Region of study	Fetal karyotype	Ultrasound features of hypospadias	Other ultrasound findings	PPV of fetal hypospadias	PPV of fetal external genital abnormality
Epelboym et al. 2017^23^	**Philips**	**Single-center, retrospective**	**English**	**32**	**NA**	**28**	**USA**	**NA**	**Blunted penile tip, chordee, short penile**	**Bifid scrotum (6), undescended testes (3), renal agenesis (1)**	**18/25 (72%)**	**21/25 (84%)**
Fuchs et al. 2019^24^	**NA**	**Single-center, retrospective**	**English**	**61**	**NA**	**23**	**Franch**	**46, XX (7/61), 46, XY (42/61)**	**Enlarged distal penile, ventral curvature, shortened urethra**	**Megalourethra (1), bilateral ureteral hydronephrosis (2), FGR (15/42, 36%)**	**29/38 (76%)**	**55/61 (90%)**
Li et al. 2019^11^	**SAMSUNG, General Electric**	**Single-center, retrospective**	**English**	**47**	**31**	**29**^**+5**^	**China**	**46, XY (27/30), 47, XXY (2/30), 46 X small Y (1/30)**	**Blunted penile tip, chordee, incomplete prepuce, short penile, abnormal urinary stream, ventral deflection**	**Urethral groove (9), penoscrotal transposition (13), short limbs (1)**	**22/27 (81%)**	**32/34 (94%)**
Zhu et al. 2020^25^	**General Electric**	**Single-center, retrospective**	**Chinese**	**31**	**26**	**26**^**+5**^	**China**	**NA**	**Small/shortened penile, blunted penile tip, chordee, abnormal urinary stream, tulip sign**	**Sex development disorder (3) buried penis (1), polycystic kidneys (1)**	**20/24 (83%)**	**27/31 (87%)**
Luo et al. 2020^26^	**General Electric**	**Single-center, retrospective**	**Chinese**	**31**	**28**	**31**^**+5**^	**China**	**NA**	**Small penile, tulip sign, blunted penile tip, urethra interruption**	**Ventricular septal defect (2), clitoromegaly (2)**	**19/23 (83%)**	**28/31 (90%)**
Zeng et al. 2020^27^	**General Electric**	**Single-center, retrospective**	**Chinese**	**260**	**29**	**31**	**China**	**NA**	**Short/small penile, tulip sign, urethra interruption**	**Penoscrotal transposition (24), sex development disorder (13)**	**158/192 (82%)**	**223/260 (86%)**
Uygur et al. 2023^13^	**General Electric**	**Single-center, retrospective**	**English**	**30**	**NA**	**NA**	**Turkey**	**46, XY (18/21), 46, XY r (1/21), 47, XX +22 (1/21), 46, XX (1/21)**	**Tulip sign, ventral penile shortening/curvature, blunted penile tip**	**Uteroplacental insufficiency (8), bifid scrotum (1), FGR (8/22, 36%)**	**22/30 (73%)**	**27/30 (90%)**
Cheng et al. 2023^28^	**SAMSUNG, General Electric**	**Single-center, retrospective**	**Chinese**	**87**	**28**	**28**^**+2**^	**China**	**NA**	**Short penile, tulip sign, penile dysmorphism, ventral curvature, blunted penile tip**	**Ventral foreskin deficiency (1), dorsal foreskin thickened (2)**	**48/50 (96%)**	**73/87 (84%)**
Abgral et al. 2024^29^	**NA**	**Single-center, retrospective**	**English**	**21**	**33**	**22**^**+5**^	**Franch**	**46, XY (16/17)** **45, X/46, XY (1/17),**	**Penile curvature/shortening, abnormal positioning of urethral meatus**	**Growth delay (5), FGR (6/21, 29%), cloacal malformation (1)**	**17/21 (81%)**	**19/21 (90%)**

Abbreviations: PPV, positive prediction value; FGR, fetal growth restriction; y, years; w, weeks. The PPVs of fetal hypospadias and fetal external genital abnormality are performed by two-dimensional ultrasound. Studies are listed in order of publication.

### Risk of bias

**Fig. 2:**
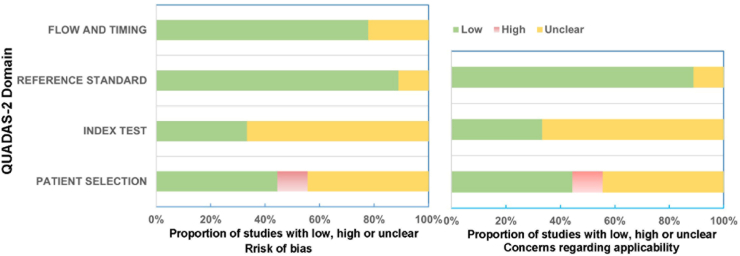
Graphical illustration of QUADAS-2 results. The proportion of studies with low, high, or unclear risk of bias and applicability concerns is shown by the QUADAS-2 domains. The QUADAS-2 scale was used to assess the risk of bias.

A thorough risk of bias assessment of the included studies was conducted using the QUADAS-2 standard. The assessment results are presented in Fig. 2, and further details can be found in Table S2. Regarding patient selection, only one out of the nine studies was found to have a high risk of bias. This study^28^ exhibited characteristics that might compromise the representativeness of the patient population. The selection process in this study had limitations, such as a small sample size and a narrow recruitment scope, which could have introduced substantial selection bias. As a result, it may lead to deviations in the research results.

### Common ultrasound manifestations in diagnosing fetal hypospadias

Common ultrasound manifestations in the diagnosis of fetal hypospadias include short/small penile, tulip sign, abnormal urinary stream, urethral interruption, and incomplete prepuce. Meanwhile, hypospadias is frequently accompanied by other anomalies, such as FGR, penoscrotal transposition, uteroplacental insufficiency, short limbs, and cryptorchidism. These accompanying abnormalities provide additional evidence for diagnosing fetal hypospadias and its related syndromes, which is significant for comprehensively evaluating fetal health. The most common ultrasound manifestations were short/small penile (95/133, 71%) and tulip sign (67/133, 50%) (including three studies),^25^^,^^26^^,^^28^ with accuracies of 86% and 79%, respectively (Table 2). The data of specific ultrasound findings were statistically obtained from three included studies.^11^^,^^13^^,^^25^

**Table 2:** Accuracy of different specific ultrasound features in diagnosing fetal hypospadias and PPVs for fetal hypospadias or external genitalia.

Specific ultrasound findings	Total number, (n)	Number of correct diagnoses, (n)	Accuracy (95% CI)
Short/small penile	54	45	0.86 (0.76–0.94)
Tulip sign	56	45	0.79 (0.73–0.90)
Blunted penile tip	27	22	0.77 (0.67–0.86)
Penile curvature	19	13	0.82 (0.65–1)
Abnormal urinary stream	24	14	0.76 (0.52–1)
Overall PPV of fetal hypospadias (95% CI)		0.81 (0.77–0.85)	
Overall PPV of fetal external genitalia (95% CI)		0.88 (0.86–0.91)	

Abbreviations: PPV, positive predictive value; CI, confidence interval. The 95% CI was calculated using the bootstrap method. The data on specific ultrasound features was statistically derived from three included studies.^11^^,^^13^^,^^25^

### Ultrasound performance for fetal hypospadias detection

**Fig. 3:**
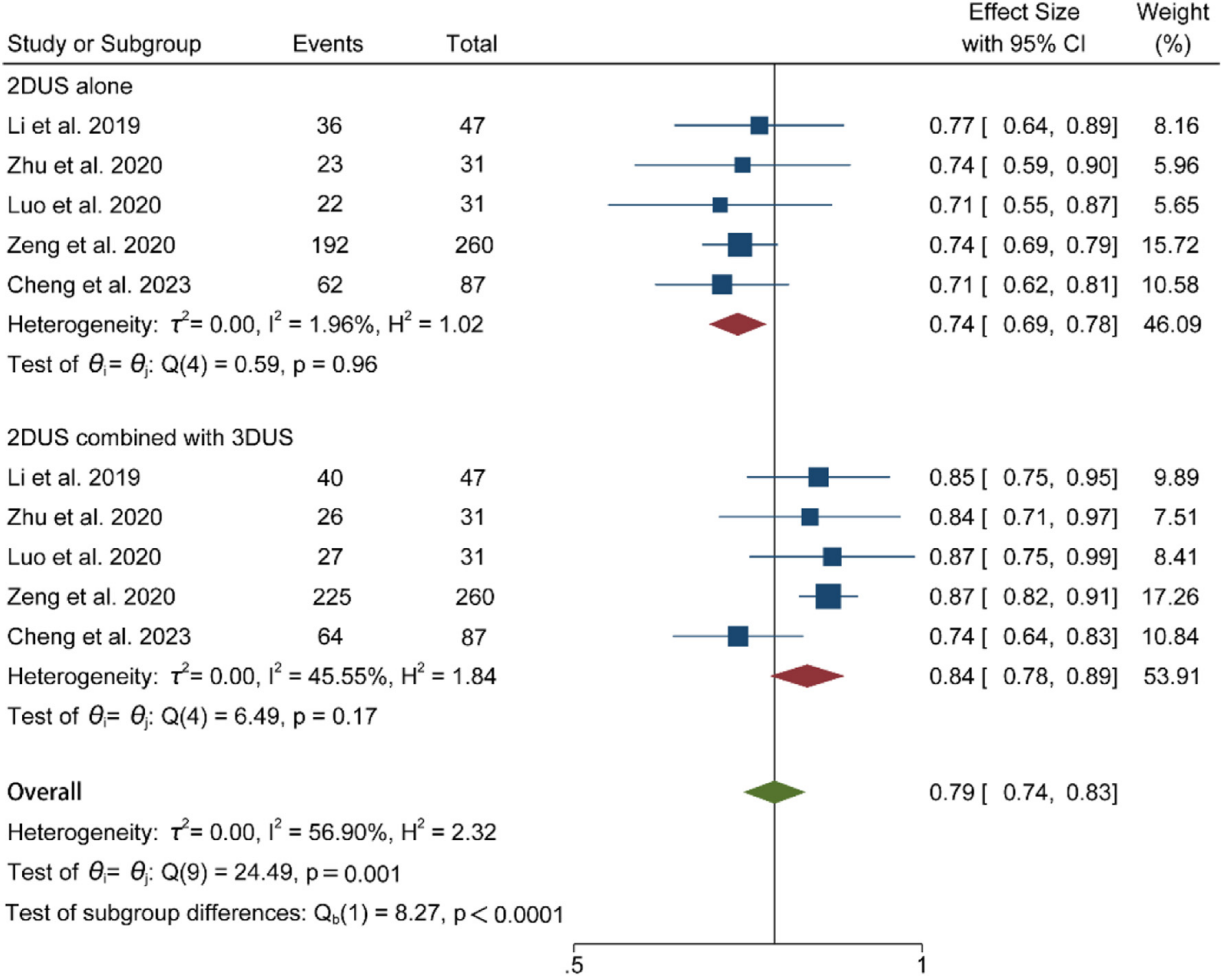
Forest plot for comparison of the accuracy of 2DUS combined with 3DUS and that of 2DUS alone (*P* < 0.0001). The diagnostic accuracy of 2DUS alone was lower than that of the combined application of 2DUS and 3DUS. Abbreviations: 2DUS, two-dimensional ultrasound; 3DUS, three-dimensional ultrasound.

**Fig. 4:**
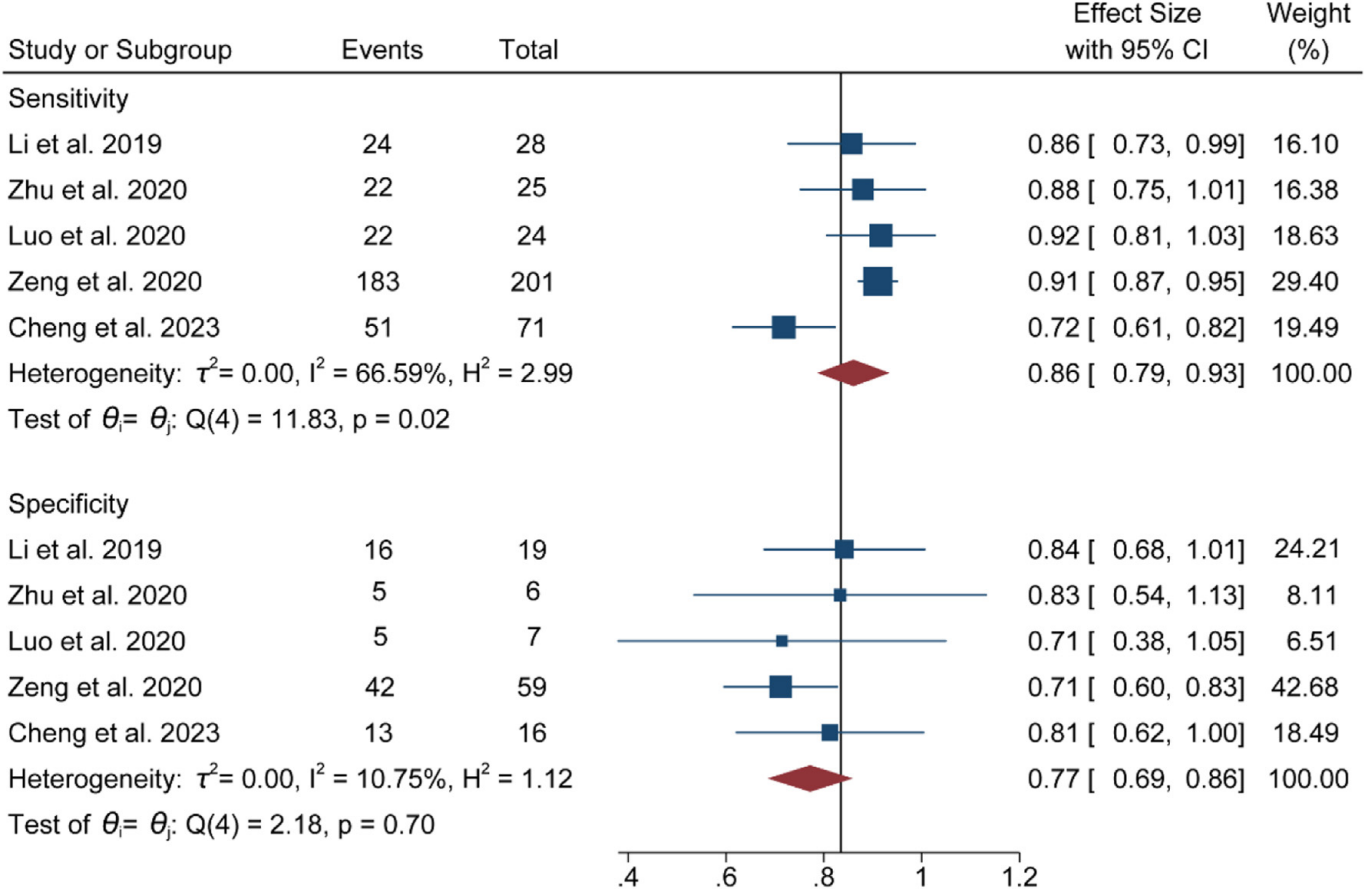
Forest plots showing the sensitivity and specificity of 2DUS combined with 3DUS for fetal hypospadias. Abbreviations: 2DUS, two-dimensional ultrasound; 3DUS, three-dimensional ultrasound.

**Fig. 5:**
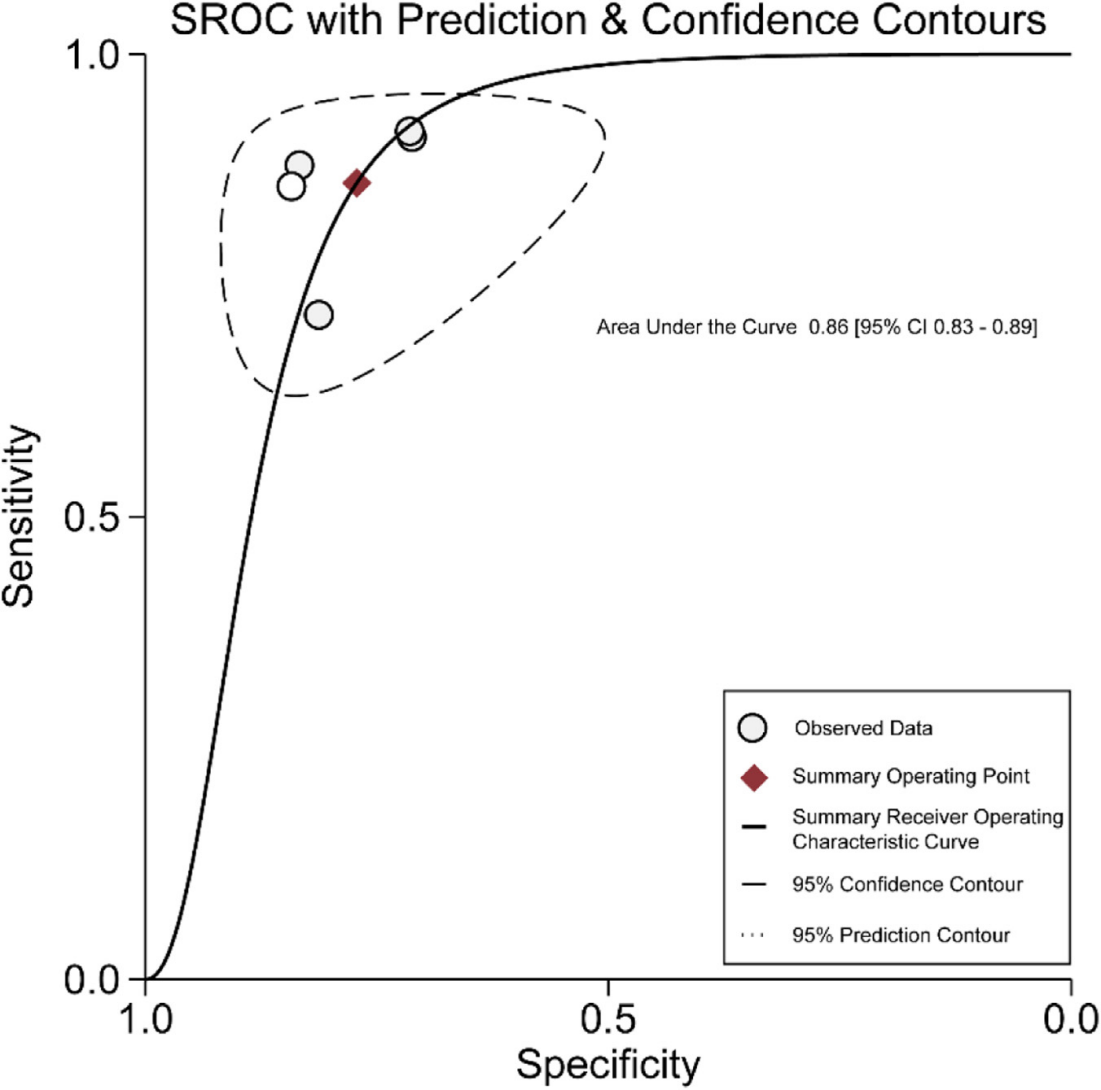
The SROC curve of 2DUS combined with 3DUS for diagnosing fetal hypospadias. The AUC was 0.86 (95% CI: 0.83–0.89), indicating that the combination of 2DUS and 3DUS had high diagnostic efficiency in diagnosing fetal hypospadias. Abbreviations: AUC, area under the curve; 2DUS, two-dimensional ultrasound; 3DUS, three-dimensional ultrasound; CI, confidence interval; SROC, summary receiver operating characteristic.

Table 2 shows that the overall PPVs of 2DUS for the diagnosis of fetal hypospadias and external genital malformations were 81% (95% CI: 77%–85%) and 88% (86%–91%), respectively. To further assess the diagnostic performance of 2DUS combined with 3DUS, we adopted multiple analytical methods and reporting strategies to calculate and analyze key indicators, following the researchers' recommendations in the medical research field.^30^, ^31^, ^32^ The statistical results of five studies showed that in diagnosing fetal hypospadias, the accuracy of the 2DUS combined with 3DUS was 84% (95% CI: 78%–89%; *I*^2^ = 45.55%; GRADE: Low), and that of 2DUS alone was 74% (95% CI: 69%–78%; *I*^2^ = 1.96%; GRADE: Low) (Fig. 3). The difference between the two methods was 10%. The sensitivity of 2DUS combined with 3DUS was 86% (95% CI: 79%–93%; *I*^2^ = 66.59%; GRADE: Low), and the specificity was 77% (95% CI: 69%–86%; *I*^2^ = 10.75%; GRADE: Low) (Fig. 4). Additionally, the AUC was 0.86, within the range of 0.8–0.9, indicating good diagnostic performance in detecting fetal hypospadias (Fig. 5). However, it should be noted that the interpretation of these results needs to consider the clinical context and potential impact on patient management decisions.

### Other findings

We further analyzed the data to assess the optimal time point for ultrasound diagnosis of fetal hypospadias. The PPV of ultrasound for diagnosing fetal hypospadias was found to be almost the same in the late stage of pregnancy (83%) than in the midgestation (80%) (Table S3). This similarity might be associated with the natural progression of fetal penile development. However, given the small sample size, the results warrant careful interpretation to avoid over-generalization. Moreover, a statistical analysis of the incidence of fetal hypospadias accompanied by FGR was conducted in three studies.^13^^,^^24^^,^^29^ The results showed that among the 85 cases of fetal hypospadias, 29 cases were concurrent with FGR (Table S4).

### Sensitivity analyses and sources of heterogeneity

To evaluate the stability and reliability of the results, the included studies were individually eliminated to observe the extent of their impact on the results. The sensitivity analysis indicated the stability of the results. Specifically, due to its higher sensitivity estimates and exact confidence intervals, the study^28^ contributed more substantially to the combined sensitivity in the overall meta-analysis, possibly resulting in a higher combined sensitivity estimate (Figure S1). As depicted in Fig. 4, the *I*^2^ for sensitivity was 66.59%, suggesting significant heterogeneity among the studies. We incorporated several variables into the meta-regression model, including languages, maternal age, and gestational age at diagnosis. Although meta-regression analysis was performed to explore potential factors, we failed to effectively identify the factors that contribute significantly to the heterogeneity of sensitivity (Figure S2). Considering that the limited number of studies may affect the robustness of the results, further confirmation in the future requires more prospective studies with large samples.

## Discussion

This study represents the first systematic evaluation of ultrasound for diagnosing fetal hypospadias. With the progression of ultrasound technology and continuous improvement in resolution, the possibility of ultrasound in the prenatal diagnosis of hypospadias is increasing in clinical practice. This study included nine eligible studies involving 600 cases, of which five were used for meta-analysis. Our findings suggest that 2DUS presented a high PPV in diagnosing fetal hypospadias and external genital malformations, but its diagnostic accuracy was lower than that of 2DUS combined with 3DUS. 2DUS combined with 3DUS showed high diagnostic performance in diagnosing fetal hypospadias, as evidenced by its sensitivity of 0.86, specificity of 0.77, and AUC of 0.86.

It is important to clarify the role of ultrasound in the diagnosis of prenatal hypospadias in clinical medical decision-making, consequently providing more precise prenatal counseling services for pregnant women and their families. Determination of fetal gender during pregnancy is a routine evaluation of the genitourinary system. It meets the parents' curiosity and provides essential information for perinatal management of gender-associated congenital anomalies.^33^ During pregnancy, as the differentiation of fetal external genitalia commences at 8–11 weeks, it is not feasible to distinguish fetal gender using ultrasound before 12 weeks of pregnancy.^34^^,^^35^ It is also not advisable to conduct ultrasound diagnosis of fetal hypospadias before 20 weeks of pregnancy. This is because the fetal urethral plate fusion process is retarded during this period, and there is a high likelihood that normal development will be misdiagnosed as hypospadias.^36^ This is also the reason why the ultrasound diagnosis of fetal hypospadias is mainly in the middle and late stages of pregnancy. Clinicians should consider multiple factors, including maternal characteristics and associated clinical indicators, rather than relying solely on the absence of difference. Larger-scale and more comprehensive studies are imperative to formulate more definitive guidelines regarding the optimal stage for ultrasound diagnosis of fetal hypospadias, thus enabling more informed clinical decision-making, efficient resource utilization, and enhanced prenatal care quality. The combination of pregnancy-associated biomarkers in maternal peripheral blood and genetic diagnosis (including mutations in genes such as Androgen Receptor, Wilms Tumor 1, and Sex-determining Region Y) may potentially bridge the gap in diagnosing fetal hypospadias during the early and middle stages of pregnancy. Studies have indicated that the levels of alpha-fetoprotein (AFP) and free beta human chorionic gonadotrophin (free beta-HCG) in the maternal serum during early and middle gestation are associated with the likelihood of fetal hypospadias.^37^^,^^38^ Hypospadias is not merely an isolated and localized developmental anomaly; it involves the whole body and is frequently accompanied by growth restriction (e.g., height and finger length), cryptorchidism, and cardiovascular diseases. Previous investigations^39^^,^^40^ have shown that maternal serum lamin A can be applied to early and middle pregnancy screening for congenital heart disease. These studies offer novel insights and approaches for the discovery of potential protein markers associated with fetal hypospadias.

In the diagnosis of fetal diseases, comprehensive utilization of various imaging methods is important for achieving an accurate diagnosis. Among them, ultrasound, as a widely used, relatively low-cost, and non-invasive examination method, plays a key role in fetal assessment.^10^ 2DUS is susceptible to numerous factors (such as fetal position, amniotic fluid volume, gestational age, and instrument resolution), and it is frequently challenging to obtain a sagittal section image of the penile, which presents difficulties in diagnosis. Nevertheless, the utilization of 2DUS combined with 3DUS can effectively compensate for the deficiencies of 2DUS in spatial display and significantly enhance the detection rate of complex structures and minute lesions. However, it is challenging to diagnose when an ultrasound image is not typical. Therefore, MRI can serve as a crucial complementary approach when ultrasound diagnosis is ambiguous or complex lesions are suspected. Compared with ultrasound, MRI is not affected by fetal bones and gas, thus possessing significant advantages in cases where ultrasound imaging is unclear because of interfering factors.^41^^,^^42^ Based on the complementary relationship between 2DUS and 3DUS, and the role of MRI as an auxiliary means when ultrasound diagnosis is limited, the integrated application of these modalities can improve the clinical diagnosis level of fetal hypospadias to some degree. The diagnostic performance of ultrasound for external genital malformations is satisfactory, but it is slightly lower in the specific diagnosis of hypospadias, which also is consistent with some retrospective studies.^13^^,^^23^^,^^24^ This indicates that the ultrasound imaging features of different types of hypospadias (e.g., glanular, coronal, penoscrotal) may overlap with those of normal GT and that subtle changes in the GT under the intricate intrauterine environment are easily overlooked. The exploration of complementary strategies between the two techniques represents an important direction for enhancing the diagnostic capability of ultrasound and improving the diagnosis of fetal hypospadias.

Additionally, our findings showed a prenatal correlation between fetal hypospadias and FGR. Three studies^13^^,^^24^^,^^29^ conducted statistical analyses of the incidence of fetal hypospadias accompanied by FGR. The results indicated that among 85 cases of fetal hypospadias, 29 cases had concurrent FGR, which suggests that the potential role of perinatal factors should not be underestimated. Some other studies also provide information that there is an increase in the frequency of hypospadias in male fetuses with FGR.^43^, ^44^, ^45^ These results highlight the crucial implications of the relationship between fetal hypospadias and FGR and underline the need for in-depth exploration of perinatal factors for better understanding, diagnosis, and management of fetal hypospadias in clinical practice. Building on this understanding, other studies have delved deeper into the perinatal factors. Their findings revealed that a series of perinatal elements, such as hypertensive disorders of pregnancy, multiple pregnancies, maternal infectious diseases, and hyperthyroidism, might be associated with fetal hypospadias and serve as potential independent risk factors for this condition.^46^, ^47^, ^48^ For pregnant women with a family history of hypospadias or those exposed to these risk factors, it is important in clinical practice to strengthen prenatal screening to identify high-risk pregnancy status at an early stage. Karyotype analysis plays an important role in diagnosing fetal hypospadias.^29^ Although it has not been widely used in existing studies, it provides numerous important supports for prenatal care of hypospadias, such as assessing prognosis, predicting fetal health, and guiding perinatal management. In the presence of a family history of genetic disorders, prenatal assessment by amniocentesis with chromosomal karyotype analysis can be an option after communicating with pregnant women.

This study had several limitations. First, there may be restrictions in promoting the study's results in routine clinical settings. The included studies did not involve essential elements, such as special ultrasound examination techniques, practical skills, equipment parameter settings, and multidisciplinary collaboration experience. Second, given the number of studies, patient selection, ultrasound equipment, and operator experience, certain aspects may undermine the reliability, representativeness, and persuasiveness of results. Unfortunately, owing to the variations in ultrasonic probe parameters and incomplete data, it is difficult to compare the ultrasonic performance differences among brands, which ultimately undermines its applicability to the general population. We found that 2DUS combined with 3DUS had higher accuracy than 2DUS alone, which is worth further evaluating in a larger population to explore its practical potential and significance. Nevertheless, before the clinical application of 2DUS combined with 3DUS, it is necessary to confirm its effectiveness compared to 2DUS and its ability to enhance prenatal diagnosis of hypospadias.

In summary, this study provides comprehensive evidence and in-depth insights that support the utilization of ultrasound technology to promote the rational application of ultrasound within the realm of prenatal diagnosis of fetal hypospadias on a broader scale and to benefit more pregnant women and fetuses. Concurrently, more prospective studies on ultrasound diagnosis of fetal hypospadias in various clinical settings should be conducted to verify and improve the existing research results and provide more comprehensive guidance for future clinical practice.

## Contributors

QZ, CZZ, and XL conceptualized the study. QZ, SHC, and CZZ developed methodology. QZ and SHC performed the literature search and abstract screening. CW and MZL extracted and assessed the data. QZ, CW, and MZL verified the underlying data. QZ, SHC, CW, MZL, HGW, and CZZ interpreted the results and prepared the tables and figures. XL supervised the entire process and helped with the discussion of the results. QZ wrote the first draft of the manuscript with input from HGW, CZZ, and XL. All authors validated, critically revised the manuscript and approved the final version of the manuscript.

## Data sharing statement

Datasets used and analyzed in the current study were obtained upon reasonable request.

## Declaration of interests

We declare no competing interests.

## References

[1] Mahboubi K., MacDonald L., Ahrens B. (2023). Geospatial analysis of hypospadias and cryptorchidism prevalence rates based on postal code in a Canadian province with stable population. J Pediatr Urol.

[2] Stukenborg J.B., Mitchell R.T., Söder O. (2021). Endocrine disruptors and the male reproductive system. Best Pract Res Clin Endocrinol Metabol.

[3] Zhang Z., Zhang Q., Liu Z. (2024). Rab25 is involved in hypospadias via the β1 integrin/EGFR pathway. Exp Cell Res.

[4] Bouty A., Ayers K.L., Pask A., Heloury Y., Sinclair A.H. (2015). The genetic and environmental factors underlying hypospadias. Sex Dev.

[5] Yu X., Nassar N., Mastroiacovo P. (2019). Hypospadias prevalence and trends in international birth defect surveillance systems, 1980-2010. Eur Urol.

[6] Kraft K.H., Shukla A.R., Canning D.A. (2010). Hypospadias. Urol Clin.

[7] Pajkrt E., Chitty L.S. (2004). Prenatal gender determination and the diagnosis of genital anomalies. BJU Int.

[8] Avni F.E., Lerisson H., Lobo M.L. (2019). Plea for a standardized imaging approach to disorders of sex development in neonates: consensus proposal from European Society of Paediatric Radiology task force. Pediatr Radiol.

[9] Meizner I., Mashiach R., Shalev J., Efrat Z., Feldberg D. (2002). The 'tulip sign': a sonographic clue for in-utero diagnosis of severe hypospadias. Ultrasound Obstet Gynecol.

[10] López Soto Á., Bueno González M., Urbano Reyes M. (2023). Imaging in fetal genital anomalies. Eur J Obstet Gynecol Reprod Biol.

[11] Li X., Liu A., Zhang Z., An X., Wang S. (2019). Prenatal diagnosis of hypospadias with 2-dimensional and 3-dimensional ultrasonography. Sci Rep.

[12] Ji E.K., Pretorius D.H., Newton R. (2005). Effects of ultrasound on maternal-fetal bonding: a comparison of two- and three-dimensional imaging. Ultrasound Obstet Gynecol.

[13] Uygur L., Sivrikoz T.S., Kalelioglu I.H. (2023). Predictive value of ultrasound in prenatal diagnosis of hypospadias: hints for accurate diagnosis. J Perinat Med.

[14] Moher D., Liberati A., Tetzlaff J., Altman D.G. (2009). Preferred reporting items for systematic reviews and meta-analyses: the PRISMA statement. BMJ.

[15] Devesa R., Muñoz A., Torrents M., Comas C., Carrera J.M. (1998). Prenatal diagnosis of isolated hypospadias. Prenat Diagn.

[16] Whiting P.F., Rutjes A.W., Westwood M.E. (2011). QUADAS-2: a revised tool for the quality assessment of diagnostic accuracy studies. Ann Intern Med.

[17] IntHout J., Ioannidis J.P., Borm G.F. (2014). The Hartung-Knapp-Sidik-Jonkman method for random effects meta-analysis is straightforward and considerably outperforms the standard DerSimonian-Laird method. BMC Med Res Methodol.

[18] Röver C., Knapp G., Friede T. (2015). Hartung-Knapp-Sidik-Jonkman approach and its modification for random-effects meta-analysis with few studies. BMC Med Res Methodol.

[19] DerSimonian R., Laird N. (1986). Meta-analysis in clinical trials. Contr Clin Trials.

[20] DeLong E.R., DeLong D.M., Clarke-Pearson D.L. (1988). Comparing the areas under two or more correlated receiver operating characteristic curves: a nonparametric approach. Biometrics.

[21] Knapp G., Hartung J. (2003). Improved tests for a random effects meta-regression with a single covariate. Stat Med.

[22] Guyatt G.H., Oxman A.D., Vist G.E. (2008). GRADE: an emerging consensus on rating quality of evidence and strength of recommendations. BMJ.

[23] Epelboym Y., Estrada C., Estroff J. (2017). Ultrasound diagnosis of fetal hypospadias: accuracy and outcomes. J Pediatr Urol.

[24] Fuchs F., Borrego P., Amouroux C. (2019). Prenatal imaging of genital defects: clinical spectrum and predictive factors for severe forms. BJU Int.

[25] Zhu Y., Wei Y., Chen S., Guo D. (2020). Prenatal ultrasound diagnosis evaluation of hypospadias. Med J West China.

[26] Luo Q., Liao L., Wang H., Tang K. (2020). The diagnostic values of three-dimensional multiplanar ultrasound in qualitative analysis of fetal hypospadias. Practical J Clin Med.

[27] Zeng Z., Li Y., Peng X., Wu X., Li C. (2020). Analysis of the value of three-dimensional ultrasound multiplanar imaging model (3DUSMI) in the qualitative diagnosis of fetal hypospadias. Electronic J Practical Gynecol Endocrinol.

[28] Cheng D. (2023). Effect of 4DUS+2DUS test on diagnostic accuracy of fetal severe hypospadias in pregnant women receiving prenatal screening. Clin Res.

[29] Abgral M., Bouvattier C., Senat M.V., Bouchghoul H. (2024). The role of pre- and postnatal investigations in suspected isolated hypospadias. J Gynecol Obstet Hum Reprod.

[30] Mansournia M.A., Nazemipour M., Etminan M. (2022). P-value, compatibility, and S-value. Glob Epidemiol.

[31] Greenland S., Mansournia M.A., Joffe M. (2022). To curb research misreporting, replace significance and confidence by compatibility: a Preventive Medicine Golden Jubilee article. Prev Med.

[32] Mansournia M.A., Nazemipour M. (2024). Recommendations for accurate reporting in medical research statistics. Lancet.

[33] Perlitz Y., Keselman L., Haddad S., Mukary M., Izhaki I., Ben-Ami M. (2011). Prenatal sonographic evaluation of the penile length. Prenat Diagn.

[34] Feldman K.W., Smith D.W. (1975). Fetal phallic growth and penile standards for newborn male infants. J Pediatr.

[35] Stephens J.D., Sherman S. (1983). Determination of fetal sex by ultrasound. N Engl J Med.

[36] Odeh M., Granin V., Kais M., Ophir E., Bornstein J. (2009). Sonographic fetal sex determination. Obstet Gynecol Surv.

[37] Chen Y., Huang J., Mei J. (2019). A risk prediction model for fetal hypospadias by testing maternal serum AFP and free beta-HCG. Clin Biochem.

[38] Schneuer F.J., Bower C., Holland A.J. (2016). Maternal first trimester serum levels of free-beta human chorionic gonadotrophin and male genital anomalies. Hum Reprod.

[39] Chen L., Gu H., Li J. (2016). Comprehensive maternal serum proteomics identifies the cytoskeletal proteins as non-invasive biomarkers in prenatal diagnosis of congenital heart defects. Sci Rep.

[40] Chen L., Xiu Y., Wu Q. (2022). Maternal serum Lamin A is a potential biomarker that can predict adverse pregnancy outcomes. eBioMedicine.

[41] Li K., Zhang X., Yan G., Zheng W., Zou Y. (2021). Prenatal diagnosis and classification of fetal hypospadias: the role and value of magnetic resonance imaging. J Magn Reson Imaging.

[42] Nemec S.F., Kasprian G., Brugger P.C. (2011). Abnormalities of the penis in utero--hypospadias on fetal MRI. J Perinat Med.

[43] Toufaily M.H., Roberts D.J., Westgate M.N., Hunt A.T., Holmes L.B. (2018). Hypospadias, intrauterine growth restriction, and abnormalities of the placenta. Birth Defects Res.

[44] Hashimoto Y., Kawai M., Nagai S. (2016). Fetal growth restriction but not preterm birth is a risk factor for severe hypospadias. Pediatr Int.

[45] Fujimoto T., Suwa T., Kabe K., Adachi T., Nakabayashi M., Amamiya T. (2008). Placental insufficiency in early gestation is associated with hypospadias. J Pediatr Surg.

[46] Wang Y., Wang L., Yang Z., Chen F., Liu Z., Tang Z. (2022). Association between perinatal factors and hypospadias in newborns: a retrospective case-control study of 42,244 male infants. BMC Pregnancy Childbirth.

[47] Jamaladin H., van Rooij I., van der Zanden L.F.M., van Gelder M., Roeleveld N. (2020). Maternal hypertensive disorders and subtypes of hypospadias: a Dutch case-control study. Paediatr Perinat Epidemiol.

[48] Sun G., Tang D., Liang J., Wu M. (2009). Increasing prevalence of hypospadias associated with various perinatal risk factors in Chinese newborns. Urology.

