# Supplementary material for: Performance of ultrasound in detecting fetal hypospadias during pregnancy: a pooled analysis
Source: eClinicalMedicine. 2025 Feb 1;81:103091. doi: 10.1016/j.eclinm.2025.103091 (PMC11840197; doi:10.1016/j.eclinm.2025.103091)
Supplement: Table S1 [file mmc1.docx]

**Table S1.** The search strategy was used to identify relevant studies using PubMed, Web of Science, Scopus, Embase, and CNKI databases. The number of studies is indicated after the database names. CNKI: China National Knowledge Infrastructure.

| **Database** | **Search Terms** | **Study Number** |
| --- | --- | --- |
| **PubMed** | (fetal and (hypospadias OR genital diseases OR penile diseases)) AND (prenatal diagnosis OR intrauterine diagnosis OR antenatal diagnosis OR prenatal screening OR antenatal screening OR fetal diagnosis OR fetal screening) AND (ultrasonography OR medical sonography OR ((ultrasonographic OR ultrasonic OR ultrasound) AND imaging) OR diagnostic ultrasound OR ultrasonic diagnosis) | 1634 |
| **Web of Science** | ((Fetal) AND (Hypospadias OR Genital Diseases OR Penile Diseases)) AND ((Fetal OR Prenatal OR Intrauterine OR Antenatal) AND (Screening OR Diagnosis)) AND (ultrasonography OR ultrasound OR ((Ultrasonic OR Ultrasonographic) AND (Imaging OR Diagnosis))) | 432 |
| **Scopus** | TITLE-ABS-KEY ((Fetal) AND (Hypospadias OR Genital Diseases OR Penile Diseases)) and ((Fetal OR Prenatal OR Intrauterine OR Antenatal) AND (Screening OR Diagnosis)) AND (ultrasonography OR ultrasound OR ((Ultrasonic OR Ultrasonographic) AND (Imaging OR Diagnosis))) | 909 |
| **Embase** | ('hypospadias' OR 'male genital system disease' OR 'penis disease') AND ('ultrasound') | 948 |
| **CNKI** | “hypospadias” and “diagnosis” | 393 |
