# Supplementary material for: Performance of ultrasound in detecting fetal hypospadias during pregnancy: a pooled analysis
Source: eClinicalMedicine. 2025 Feb 1;81:103091. doi: 10.1016/j.eclinm.2025.103091 (PMC11840197; doi:10.1016/j.eclinm.2025.103091)
Supplement: Table S2 [file mmc2.docx]

**Table S2.** The results of the QUADAS-2 scale.

| **Study** | **Risk of bias** | | | | **Applicability concerns** | | |
| --- | --- | --- | --- | --- | --- | --- | --- |
|  | **Patient selection** | **Index test** | **Reference standard** | **Flow**  **and timing** | **Patient selection** | **Index test** | **Reference standard** |
| Epelboym et al. 2017^1^ | 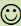 | 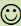 | 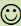 | 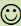 | 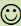 | 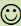 | 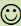 |
| Fuchs et al. 2019^2^ | 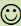 | 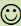 | 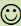 | 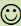 | 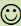 | 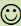 | 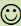 |
| Li et al. 2019^3^ | 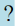 | 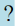 | 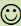 | 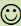 | 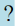 | 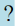 | 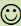 |
| Zhu et al. 2020^4^ | 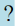 | 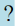 | 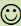 | 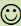 | 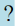 | 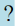 | 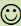 |
| Luo et al. 2020^5^ | 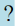 | 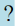 | 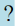 | 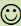 | 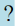 | 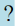 | 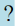 |
| Zeng et al. 2020^6^ | 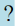 | 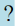 | 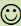 | 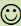 | 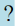 | 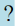 | 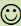 |
| Uygur et al. 2023^7^ | 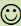 | 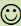 | 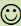 | 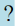 | 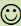 | 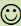 | 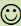 |
| Cheng et al. 2023^8^ | 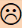 | 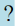 | 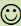 | 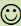 | 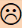 | 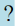 | 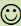 |
| Abgral et al. 2024^9^ | 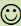 | 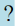 | 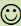 | 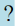 | 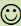 | 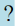 | 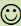 |


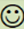
 Low-Risk
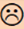
 High-Risk
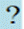
 Unclear Risk Abbreviation: QUADAS-2, Quality Assessment of Diagnostic Accuracy Studies-2.

References

1. Epelboym Y, Estrada C, Estroff J. Ultrasound diagnosis of fetal hypospadias: Accuracy and outcomes. *J Pediatr Urol* 2017; **13**(5): 484.e1-.e4.

2. Fuchs F, Borrego P, Amouroux C, et al. Prenatal imaging of genital defects: clinical spectrum and predictive factors for severe forms. *BJU Int* 2019; **124**(5): 876-82.

3. Li X, Liu A, Zhang Z, An X, Wang S. Prenatal diagnosis of hypospadias with 2-dimensional and 3-dimensional ultrasonography. *Sci Rep* 2019; **9**(1): 8662.

4. Zhu Y, Wei Y, Chen S, Guo D. Prenatal ultrasound diagnosis evaluation of hypospadias. *Med J West China* 2020; **32**(04): 584-7.

5. Luo Q, Liao L, Wang H, Tang K. The diagnostic values of three-dimensional multiplanar ultrasound in qualitative analysis of fetal hypospadias. *Practical Journal of Clinical Medicine* 2020; **17**(04): 215-7.

6. Zeng Z, Li Y, Peng X, Wu X, Li C. Analysis of the value of three-dimensional ultrasound multiplanar imaging model (3DUSMI) in the qualitative diagnosis of fetal hypospadias. *Electronic Journal of Practical Gynecologic Endocrinology* 2020; **7**(29): 130-1.

7. Uygur L, Sivrikoz TS, Kalelioglu IH, et al. Predictive value of ultrasound in prenatal diagnosis of hypospadias: hints for accurate diagnosis. *J Perinat Med* 2023; **51**(7): 932-9.

8. Cheng D. Effect of 4DUS+2DUS Test on Diagnostic Accuracy of Fetal Severe Hypospadias in Pregnant Women Receiving Prenatal Screening. *Clinical Research* 2023; **31**(11): 137-40.

9. Abgral M, Bouvattier C, Senat MV, Bouchghoul H. The role of pre- and postnatal investigations in suspected isolated hypospadias. *J Gynecol Obstet Hum Reprod* 2024; **53**(7): 102781.
