# Supplementary material for: Performance of ultrasound in detecting fetal hypospadias during pregnancy: a pooled analysis
Source: eClinicalMedicine. 2025 Feb 1;81:103091. doi: 10.1016/j.eclinm.2025.103091 (PMC11840197; doi:10.1016/j.eclinm.2025.103091)
Supplement: Table S3 [file mmc3.docx]

**Table S3.** The PPV of 2DUS in the diagnosis of fetal hypospadias in the middle and late stages of pregnancy.

| Pregnant stages | References | Median gestational age at diagnosis (w) | Mean gestational age at diagnosis (w) (± SD) | PPV of fetal hypospadias | Overall PPV |
| --- | --- | --- | --- | --- | --- |
| Middle | Fuchs et al. 2019^1^ | 23 | 24.14 ± 2.23 | 29/38(76%) | 80% |
|  | Zhu et al. 2020^2^ | 26^+5^ |  | 20/24(83%) |  |
|  | Abgral et al. 2024^3^ | 22^+5^ |  | 17/21(81%) |  |
| Late | Epelboym et al. 2017^4^ | 28 | 29.74 ± 1.63 | 18/25(72%) | 83% |
|  | Li et al. 2019^5^ | 29^+5^ |  | 22/27(81%) |  |
|  | Luo et al. 2020^6^ | 31^+5^ |  | 19/23(83%) |  |
|  | Zeng et al. 2020^7^ | 31 |  | 158/192(82%) |  |
|  | Cheng et al. 2023^8^ | 28^+2^ |  | 48/50(96%) |  |

Abbreviations: PPV, positive predictive value; 2DUS, two-dimensional ultrasound. Stata software calculated the pooled mean gestational age at diagnosis and overall PPV (StataCorp, College Station, TX, USA). The references were obtained from the eight studies.^1-8^

References

1. Fuchs F, Borrego P, Amouroux C, et al. Prenatal imaging of genital defects: clinical spectrum and predictive factors for severe forms. *BJU Int* 2019; **124**(5): 876-82.

2. Zhu Y, Wei Y, Chen S, Guo D. Prenatal ultrasound diagnosis evaluation of hypospadias. *Med J West China* 2020; **32**(04): 584-7.

3. Abgral M, Bouvattier C, Senat MV, Bouchghoul H. The role of pre- and postnatal investigations in suspected isolated hypospadias. *J Gynecol Obstet Hum Reprod* 2024; **53**(7): 102781.

4. Epelboym Y, Estrada C, Estroff J. Ultrasound diagnosis of fetal hypospadias: Accuracy and outcomes. *J Pediatr Urol* 2017; **13**(5): 484.e1-.e4.

5. Li X, Liu A, Zhang Z, An X, Wang S. Prenatal diagnosis of hypospadias with 2-dimensional and 3-dimensional ultrasonography. *Sci Rep* 2019; **9**(1): 8662.

6. Luo Q, Liao L, Wang H, Tang K. The diagnostic values of three-dimensional multiplanar ultrasound in qualitative analysis of fetal hypospadias. *Practical Journal of Clinical Medicine* 2020; **17**(04): 215-7.

7. Zeng Z, Li Y, Peng X, Wu X, Li C. Analysis of the value of three-dimensional ultrasound multiplanar imaging model (3DUSMI) in the qualitative diagnosis of fetal hypospadias. *Electronic Journal of Practical Gynecologic Endocrinology* 2020; **7**(29): 130-1.

8. Cheng D. Effect of 4DUS+2DUS Test on Diagnostic Accuracy of Fetal Severe Hypospadias in Pregnant Women Receiving Prenatal Screening. *Clinical Research* 2023; **31**(11): 137-40.
