# Supplementary material for: Performance of ultrasound in detecting fetal hypospadias during pregnancy: a pooled analysis
Source: eClinicalMedicine. 2025 Feb 1;81:103091. doi: 10.1016/j.eclinm.2025.103091 (PMC11840197; doi:10.1016/j.eclinm.2025.103091)
Supplement: Table S4 [file mmc4.docx]

**Table S4.** Statistics of three studies on the incidence of FGR in fetal hypospadias.

| References | Incidence of FGR in hypospadias | Total incidence |
| --- | --- | --- |
| Fuchs et al. 2019^1^ | 15/42 (36%) | 34% |
| Uygur et al. 2023^2^ | 8/22 (36%) |  |
| Abgral et al. 2024^3^ | 6/21 (29%) |  |

FGR: fetal growth restriction. The total incidence was calculated using Stata statistical software (StataCorp, College Station, TX, USA). The included studies were derived from the three studies.^1-3^

References

1. Fuchs F, Borrego P, Amouroux C, et al. Prenatal imaging of genital defects: clinical spectrum and predictive factors for severe forms. *BJU Int* 2019; **124**(5): 876-82.

2. Uygur L, Sivrikoz TS, Kalelioglu IH, et al. Predictive value of ultrasound in prenatal diagnosis of hypospadias: hints for accurate diagnosis. *J Perinat Med* 2023; **51**(7): 932-9.

3. Abgral M, Bouvattier C, Senat MV, Bouchghoul H. The role of pre- and postnatal investigations in suspected isolated hypospadias. *J Gynecol Obstet Hum Reprod* 2024; **53**(7): 102781.
