# Supplementary material for: Performance of ultrasound in detecting fetal hypospadias during pregnancy: a pooled analysis
Source: eClinicalMedicine. 2025 Feb 1;81:103091. doi: 10.1016/j.eclinm.2025.103091 (PMC11840197; doi:10.1016/j.eclinm.2025.103091)
Supplement: Figure S1 [file mmc5.docx]

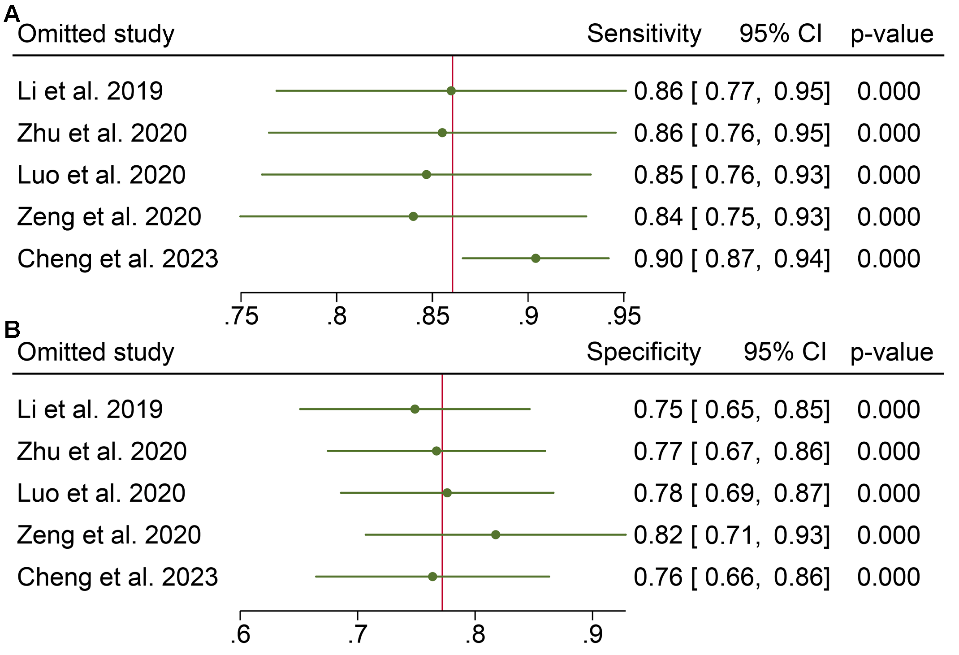


**Figure S1.** The influence of each study on the outcome of the meta-analysis. (A-B) Forest plots for sensitivity analysis of sensitivity and specificity. Sensitivity analyses excluded individual studies to determine the results' robustness.
