# Supplementary material for: Performance of ultrasound in detecting fetal hypospadias during pregnancy: a pooled analysis
Source: eClinicalMedicine. 2025 Feb 1;81:103091. doi: 10.1016/j.eclinm.2025.103091 (PMC11840197; doi:10.1016/j.eclinm.2025.103091)
Supplement: Figure S2 [file mmc6.docx]

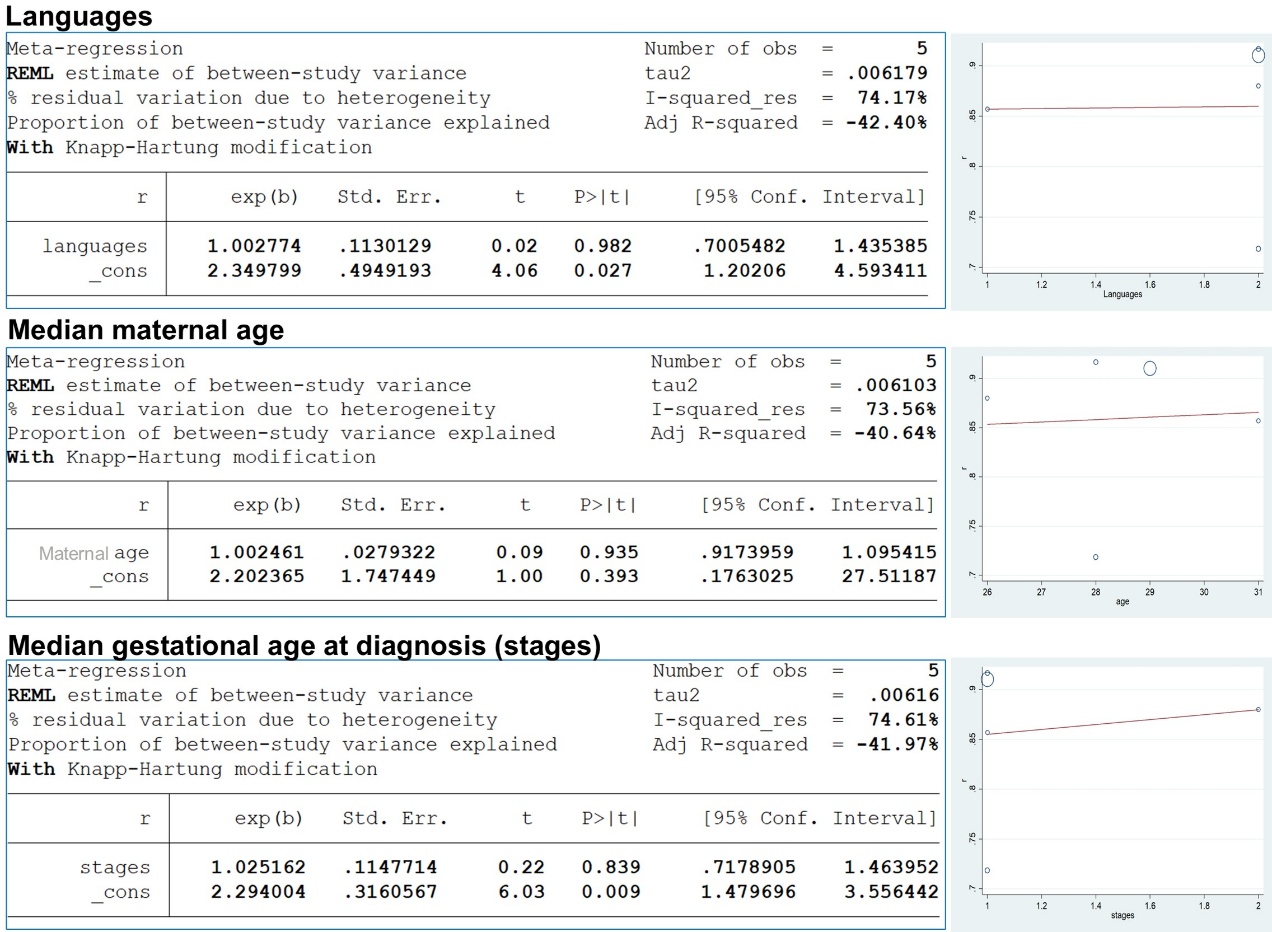


**Figure S2.** Meta-regression analysis of factors affecting diagnostic test sensitivity. The variables (languages, median maternal age, and median gestational age at diagnosis) did not significantly affect the sensitivity.
